# Potentially Inappropriate Medication Use in Older Hospitalized Patients with Type 2 Diabetes: A Cross-Sectional Study

**DOI:** 10.3390/pharmacy8040219

**Published:** 2020-11-17

**Authors:** Rishabh Sharma, Manik Chhabra, Kota Vidyasagar, Muhammed Rashid, Daniela Fialova, Akshaya S. Bhagavathula

**Affiliations:** 1 Department of Pharmacy Practice, Indo-Soviet Friendship College of Pharmacy, Moga 142001, Punjab, India; rishabhsharma19144@gmail.com (R.S.); manikchhabra57@gmail.com (M.C.); 2 Department of Pharmaceutical Sciences, University College of Pharmaceutical Sciences, Hanamkonda 506009, Telangana, India; vidyasagarkota2@gmail.com; 3 Department of Pharmacy Practice, Sri Adichunchanagiri College of Pharmacy, Adichunchanagiri University, B G Nagara 571448, Karnataka, India; muhammedrashid2@gmail.com; 4 Department of Social and Clinical Pharmacy, Faculty of Phamacy, 50005 Hradec Králové, Czech Republic; fialovad@faf.cuni.cz; 5 Department of Geriatrics and Gerontology, 1st Faculty of Medicine, Charles University, 12000 Prague, Czech Republic

**Keywords:** potentially inappropriate medications, older patients, Beers Criteria, comorbidities

## Abstract

**Background:** Older patients with type 2 diabetes mellitus (T2DM) are at greater risk of receiving potentially inappropriate medications (PIM) during hospitalization which may result in adverse outcomes. **Aim:** To evaluate the extent of PIM use in the older population with T2DM during hospitalization in a tertiary care hospital in India. **Methods:** A cross-sectional study was carried out from August 2019 to January 2020 in a tertiary care teaching hospital among the older population (aged ≥ 65 years) hospitalized with T2DM. Medications prescribed during hospitalization were reviewed following Beers Criteria 2019 to identify the extent of polypharmacy and PIM use. Binary logistic regression was applied to determine the factors associated with PIM use. **Results:** The mean age of the 150 patients hospitalized with T2DM was 68.85 ± 5.51 years, most of whom were men (54.7%). The participants had at least four comorbidities and were receiving an average of nine medications per day; the median length of hospital stay was 8 days (interquartile range (IQR): 4–19 days). Overall, three quarters (74%) of the participants had at least one PIM prescribed during their hospitalization as per Beers Criteria. Significant factors associated with the use of PIM during hospitalization are patients taking a higher number of medications (odds ratio (OR): 7.85, 95% CI 1.49–41.10), lower creatinine clearance values (OR: 12.90, 95% CI 2.81–59.28) and female patients (OR: 2.29; 95% CI: 1.05–4.97). **Conclusions:** PIM use is frequently observed in older T2DM patients during hospitalization. Polypharmacy, reduced renal function and female gender are associated with higher PIM use. Engaging clinical pharmacists in evaluating medication appropriateness can improve the outcomes of older patients.

## 1. Introduction

Increased aging among the Indian population is considered as one of the driving forces behind the increased prevalence of type 2 diabetes mellitus (T2DM) in India [1,2]. Due to the escalating older adult population, it is predicted that the number of T2DM patients will rise to 79.4 million in 2030, more than half of the T2DM patients will be above 60 years of age and the World Health Organisation (WHO) has declared India as the “Diabetic Capital of the World” [3,4].

Older patients with T2DM often tend to have multiple comorbidities and complications (65–80%) namely hypertension, cardiovascular disease, retinopathy, nephropathy, neuropathy, etc. [5]. Subsequently, older patients are more likely to be on “polypharmacy (5–9 medications per day) or hyper polypharmacy (≥10 medications per day)” in order to treat their comorbidities. Polypharmacy and multiple comorbidities make older patients more vulnerable to being prescribed potentially inappropriate medications (PIMs) [6]. PIMs, are described as a medications whose adverse risks exceed their health benefits, mainly when equal or safer alternatives are available [7]. PIM use in the older population is associated with increased morbidity and mortality, increased incidence of drug-related problems, adverse drug events, prolonged hospitalization, and an increased economic burden [8,9]. In addition, several physiological and pathological changes have been observed during the aging process which sometimes precipitate drug toxicity and predispose older patients to develop adverse drug reactions [10]. Thus, pharmacotherapy requires comprehensive evaluation in older patients with T2DM, particularly during their hospitalization, in order to reduce the use of PIM and the incidence of drug toxicity.

Several international geriatric societies have established and tested specific standards and recommendations to recognize PIM use in older patients [11]. These criteria and guidelines assist health care professionals in preventing drug-related problems, drug-drug interactions, and adverse drug events associated with PIM use. Among the available worldwide criteria, Beers Criteria is one of the most reliable and widely applied globally and is one of the most explicit (developed from an extensive literature review) for determining PIM use in the older population [12,13]. The Screening Tool of Older Persons’ Prescriptions (STOPP) and Screening Tool to Alert to Right Treatment (START) are also commonly used criteria to determine PIM prescribing [14]. However, STOPP/START criteria have proven to be less useful for frail patients who suffer from illness [14], whereas Beers Criteria can be applied to the older population, regardless of frailty level. Up to now, Beers Criteria has been revised four times by Mark Beers and three times since the beginning of 2012 by the American Geriatric Society (AGS). The AGS unveiled its latest update to Beers Criteria in 2019. However, no studies have applied the updated 2019 Beers Criteria to identify PIM use in older patients with T2DM, and few international studies have been conducted to determine the extent of PIM use in the older population with T2DM [15,16,17,18]. A study in Spain identified a high prevalence of PIM use (68.1%) using STOPP criteria in older hospitalized T2DM patients, but no such studies have been conducted in the Indian population. Therefore, the aim of this study was to evaluate the extent of PIM use during hospitalization among the older population with T2DM in India using the AGS updated Beers Criteria 2019.

## 2. Materials and Methods

### 2.1. Study Design and Sample Population

A cross-sectional study was conducted from August 2019 to January 2020 in a tertiary care teaching hospital in India. The study included older patients aged ≥ 65 years with T2DM, hospitalized due to various health complications in the internal medicine ward.

We included those older patients who provided written informed consent, admitted for more than one day, and with underlying T2DM. Patients aged < 65 years of age, those who were not diagnosed with T2DM, patients with type 1 diabetes, and those who were not willing to give informed consent were excluded from the study.

### 2.2. Data Collection

Patient demographic details and clinical data such as patient history, the presence of different comorbidities, prescribed medications hospital length of stay, laboratory test reports, and radiological examinations were obtained from the patient medical record, using a pre-designed data collection form. The prescriptions were analyzed to assess the prevalence of PIM use. PIM use was identified according to the AGS updated Beers Criteria 2019. Beers Criteria is well established and has been used for the past two decades; it refers to the general population aged over 65 years regardless of their degree of frailty or residence position. Creatinine clearance (CrCl) for all the study participants was calculated with the help of the Cockcroft-Gault equation, based on their serum creatinine reported at the time of admission [19].

### 2.3. Ethical Approvals

The study was conducted in accordance with the Ethical guidelines for biomedical research on human participants by the Indian Council for Medical Research (ICMR) after obtaining approval from the Institutional Ethics Committee (IEC) (ECR/296/Indt/PB/2018/ISFCP/118).

### 2.4. Statistical Analysis

All collected data was analyzed using Stata 16 (Stata Corp) and Statistical Package for the Social Science (SPSS) free version 24 [20]. Categorical and continuous variables such as age, gender, creatinine clearance, number of medications during a hospital stay, number of diagnoses, and length of hospital stay are presented as numbers with percentage, mean with standard deviations (SD), or median with interquartile range (IQR). The predictors of PIM prescribing, including socio-demographic variables such as age, gender, number of medications, length of hospital stay, and creatinine clearance of older patients, were assessed using binary logistic regression. The odds ratio (OR) with corresponding 95% confidence interval (CI) was used to identify the factors associated with PIM use in older patients with T2DM. A *p*-value of < 0.05 was considered statistically significant.

## 3. Results

### 3.1. Sociodemographic and Clinical Characteristics

A total of 150 older patients with T2DM were included, of which 82 (54.7%) were men and 68 (45.3%) women; most of the older patients 95 (63.3%) belonged to the 65–70 years age group, and the median age of the patients was 65 years (IQR 65–90 years). It was observed that more than half, 87 (58%), of the older patients were illiterate. The sociodemographic details and clinical characteristics of the older patients and the extent of PIMs observed are described in Table 1.

The median number of diagnoses was four (IQR 1–7 diagnosis); 85 (56.7%) patients had four or more diagnoses, and 44 (29.3%) had three diagnoses. In the study, acute or chronic kidney disease was the most common comorbid condition found in association with T2DM; 59 patients had kidney disease, 74 had hypertension and 26 had a cerebrovascular accident. Table 2 describes the comorbid conditions and PIMs found in older patients along with T2DM.

The median length of hospital stay was 8 days (IQR 4–19). Out of 150 patients, 81 (54%) were on polypharmacy, and 62 (41.3%) were on hyper polypharmacy. The median number of medications per day during hospital stay was nine (IQR 3–19). It has been clarified in Beers Criteria that a few of the medications should either be avoided completely or the dosage can be adjusted after monitoring kidney function or creatinine clearance. Creatinine clearance (CrCl) was calculated with the help of the Cockcroft-Gault equation and the median CrCl was found to be 28 mL/min (IQR 4–183). More than half the patients, 84 (54.7%), had CrCl > 30 mL/min. It was observed that patients with severe renal impairment were more commonly prescribed PIMs.

### 3.2. Prevalence of PIM

According to Beers Criteria 2019, 111 (74%) patients received at least one PIM. The details of the overall prevalence of PIM use in this study are shown in Table 3. Overall, 58 (38.7%) patients were prescribed at least one PIM, 37 (24.7%) were prescribed two PIMs, and 16 (10.7%) were prescribed ≥ three PIMs as shown in Table 1. In our study, the most commonly prescribed PIMs were human insulin according to random blood sugar, proton pump inhibitors, glimepiride, enoxaparin and ranitidine.

### 3.3. Important Predictors of PIM Prescribing

The factors associated with PIM prescribing are summarised in Table 4. Using binary logistic regression, we found that older women with T2DM were associated with a 129% higher chance than men of being exposed to PIMs (OR: 2.29, 95% CI 1.05–4.97, *p* = 0.036). Increased number of drugs use (≥10 medications per day) was also associated with an increased risk of PIMs (OR: 7.85, 95% CI 1.49–41.10, *p* = 0.015). Reduced renal clearance (CrCL < 30 mL/min) was associated with a higher chance of having PIMs than in those with normal renal function (CrCL > 120 mL/min).

## 4. Discussion

The increased prevalence of PIM is a significant health concern arising in older Indian adults because of its association with adverse health outcomes, which demand immediate attention [21]. To our knowledge, this is the first study to investigate the prevalence of PIM in older Indian patients with T2DM using the latest version of the internationally accepted Beers Criteria 2019. The analysis of the criteria was conducted by the clinical pharmacists on older hospitalized T2DM patients with multiple comorbidities and polypharmacy in a tertiary care hospital in India. Our study found that three quarters of older patients were prescribed at least one PIM during their hospital stay. Moreover, the results show that T2DM is more prevalent among the 65–70 years age group. The results of our study are similar to the recent study that applied 2019 Beers Criteria on patients aged 65–74 years attending primary care centers in Spain and which reported a PIM prevalence of 68.8% [22]. Furthermore, another recent study by Sharma et al. reported that nearly 62% of older patients aged 65–70 years received at least one PIM during hospitalization [23]. To date, there are no data available on T2DM patients using the latest version of Beers Criteria 2019 with which to make any comparison. The data from previous studies using the 2015 version in older hospitalized patients with diabetes, reported 54.5% of patients to have at least one Beers-listed PIM in Spain [18], and 56% in Canada [24]. This high prevalence could be because older patients with T2DM have more comorbidities than the general population and therefore they receive a high number of medications that increase their risk of PIM prescribing.

In our study, more than 90% of older patients with T2DM were either on polypharmacy (5–9 drugs per day) or hyper polypharmacy (≥10 drugs per day) during their hospital stay. Moreover, all of the patients with T2DM had two or more comorbidities. Older patients with T2DM are usually required to take multiple drug regimens to control glycaemic condition and T2DM-associated complications [25]. The average number of drugs prescribed per prescription during a hospital stay was nine, which is on the higher side compared to the previous studies done on older patients in India (eight), and the USA (eight) [26,27,28]. The results show that hypertension (49.3%) was the most common comorbidity in older patients with T2DM. The results are compared with various research studies that showed hypertension to be the primary cause of morbidity in the older population with T2DM [23].

Among antidiabetic medications, the most commonly prescribed PIMs were short-acting insulin according to the sliding scale, and glimepiride, since these drugs can cause severe prolonged hypoglycemia in older adults, whereas short or rapid-acting insulin, according to the sliding scale, is an agent approved for diabetic patients. However, older patients may have a higher risk of hypoglycemia without improving hyperglycemia management [24].

Overall, proton pump inhibitors (PPIs) and clonazepam were the most commonly prescribed PIMs independent of diagnosis category. PPIs, e.g., pantoprazole, rabeprazole, and omeprazole, are approved for reducing gastric acid production and are the most commonly prescribed medications in the hospital setting. PPI use for more than eight weeks is deemed inappropriate and should be avoided in older adults as per Beers Criteria 2019 since it is associated with the risk of clostridium difficile infection and increased probability of bone loss and fractures [29,30,31]. Benzodiazepine use is widespread among older adults but should be avoided since this medication increases cognitive impairment, delirium, falls and fractures [32].

Our study identified two drug-drug interactions that should be avoided in older adults based on Beers Criteria 2019. Despite a clear indication that certain medications should either be avoided or their dosage reduced according to the varying kidney function of the older patient, we have identified eight medications from this category that have been prescribed to 37 patients with impaired kidney function or low creatinine clearance. Enoxaparin, ranitidine, and dabigatran were prescribed at the usual dose in older diabetic patients, despite a decrease in creatinine clearance.

The main factors associated with PIM use were female sex, polypharmacy, and reduced renal function. Two systematic reviews reported that women and those using a large number of medications were at a higher risk for PIM use in acute care, long-term care and in community settings [33,34]. Several studies have shown that female gender, polypharmacy, and hyper polypharmacy are risk factors for PIM use, as women consult health professionals more often and consume more medications than men [35]. Impaired renal function is widely reported in the older population, particularly T2DM patients, and therefore an alternative medication or dosage reduction is recommended, no matter the criteria used [11,12,13,14].

Our study also highlights the failure to implement Beers Criteria in geriatric health care even in tertiary care hospitals in India. The AGS mentions that Beers Criteria can serve as a tool to evaluate the quality of care, so we can conclude that PIMs are causing more harm to older diabetic patients’ quality of life and increasing the economic burden. There is a need to create awareness about Beers Criteria amongst patients and physicians to justify its use and improve geriatric health care. There is also a need for the regulatory authorities to take necessary steps regarding the mandatory implementation of Beers Criteria. This study also demonstrates that creatinine clearance rate should be monitored carefully, and appropriate dose modifications made while prescribing medications in older patients. Furthermore, PIM use was associated with increased risk of adverse drug events, and their extent is concerning in developing countries and should be corroborated by further studies.

This study has several strengths and limitations. To our knowledge, this is the first study to determine the extent of PIM use and related complications based on Beers Criteria 2019 in older patients hospitalized with T2DM in India. The study limitations are, firstly, that the drug data was collected from a specific institution for all study participants and therefore the study findings may not be universally applicable to all older adults with T2DM. Secondly, many older diabetic patients were taking other traditional medications before their admission and during their hospital stay. Third, limited sample size and cross-sectional study design did not allow evaluation of the medication appropriateness at an individual level. Thus, we were only able to screen the populations at risk. Finally, we used widely applied Beers Criteria to determine the PIMs exposure. It is important to note that we cannot imply that inappropriate medication use is necessarily linked to negative outcomes, and under some circumstances some “inappropriate” medication might be appropriately indicated.

## 5. Conclusions

PIMs are frequently used in older T2DM patients during their hospitalization. Polypharmacy, reduced renal function and female gender are associated with higher PIM use. Engaging clinical pharmacists in evaluating medication appropriateness can improve older patients’ outcomes.

## Figures and Tables

**Table 1:** Characteristics of the older patients with T2DM (N=150).

Characteristics of Subjects	Total (%)	Potential Inappropriate Medication Use	
		Yes	No
*Gender*			
Male	82 (54.7)	55 (67.0)	27 (33.0)
Female	68 (45.3)	56 (82.3)	12 (17.7)
*Age (years)*			
65–70	95 (63.3)	74 (77.8)	21 (22.2)
71–75	28 (18.7)	20 (71.4))	8 (28.6)
76–80	16 (10.7)	10 (62.5)	6 (37.5)
≥ 81	11 (7.3)	7 (63.6)	4 (36.4)
*Education qualification*			
Illiterate	87 (58)	66 (75.8)	21 (24.2)
Less than 5th grade	28 (18.7)	20 (71.4)	8 (28.6)
5th–10th grade	8 (5.3)	6 (75.0)	2 (25.0)
11th–12th grade	21 (14)	16 (76.1)	5 (23.9)
Undergraduate	6 (4)	3 (50.0)	3 (50.0)
*Smoking*			
Chronic regular smoker	24 (16)	14 (58.4)	10 (41.6)
Ex-smoker	12 (8)	10 (83.4)	2 (16.6)
Non-smoker	114 (76)	87 (76.4)	27 (23.6)
*Alcohol use*			
Regular current alcoholic	27 (18)	19 (70.4)	8 (29.6)
Ex alcoholic	8 (5.3)	5 (62.5)	3 (37.5)
Occasional alcoholic	7 (4.7)	4 (57.1)	3 (42.9)
Non-alcoholic	108 (72)	83 (76.8)	25 (23.2)
*Number of comorbidities*			
2	21 (14)	15 (71.4)	6 (28.5)
3	44 (29.3)	36 (81.8)	8 (18.2)
≥ 4	85 (56.7)	60 (70.5)	25 (29.5)
*Length of hospital stay (in days)*			
1–4	7 (4.7)	4 (57.1)	3 (42.9)
5–9	87 (58)	65 (74.7)	22 (25.3)
≥10	56 (37.3)	42 (75.0)	14 (25.0)
*Number of medications during a hospitalization*			
1–4	7 (4.7)	3 (42.9)	4 (57.1)
5–9	81 (54)	55 (67.9)	26 (32.1)
≥ 10	62 (41.3)	53 (85.4)	9 (14.6)
*Creatinine clearance (mL/min)*			
<30	82 (54.7)	71 (86.5)	11 (13.5)
31–60	29 (19.3)	20 (68.9)	9 (31.1)
61–90	30 (20)	17 (56.6)	13 (43.4)
91–120	9 (6)	3 (33.3)	6 (66.6)

**Table 2:** Prevalence of comorbid conditions in older patients with T2DM.

Diagnosis	Total (%)	With PIM Use	Without PIM Use
Hypertension	74 (49.3)	52 (70.2)	22 (29.8)
Cerebrovascular accident	26 (17.3)	14 (53.8)	12 (46.2)
Kidney disease	59 (39.3)	49 (83.0)	10 (17.0)
Acute febrile illness	42 (28.0)	22 (52.4)	20 (47.6)
Liver disease	39 (26.0)	24 (61.5)	15 (38.5)
Myocardial infarction	22 (14.6)	14 (63.6)	8 (36.4)
Dilated cardiomyopathy	13 (8.6)	7 (53.8)	6 (46.2)
Coronary artery disease	26 (17.3)	15 (57.6)	11 (42.4)
LV dysfunction	6 (4.0)	4 (66.6)	2 (33.4)
Congestive heart failure	12 (8.0)	8 (66.6)	4 (33.4)
Acute coronary syndrome	5 (3.3)	2 (40.0)	3 (60.0)
Anemia	24 (16.0)	10 (41.6)	14 (58.4)
Respiratory disease	14 (9.3)	6 (42.8)	8 (57.2)
Psychiatric disorder	16 (10.6)	11 (68.8)	5 (31.2)
Upper gastrointestinal bleeding	8 (5.3)	4 (50.0)	4 (50.0)
Meningitis	6 (4.0)	2 (33.4)	4 (66.6)
Urinary tract infection	8 (5.3)	7 (87.5)	1 (12.5)
Hepatorenal syndrome	9 (6.0)	7 (77.8)	2 (22.2)
Metabolic encephalopathy	12 (8.0)	5 (41.6)	7 (58.4)
Benign prostate hyperplasia	11 (7.3)	4 (36.4)	7 (63.6)
Obstructive uropathy	8 (5.3)	5 (62.5)	3 (37.5)
Vertigo	14 (9.3)	8 (57.1)	6 (42.9)
Others	42 (28.0)	16 (38.0)	26 (62.0)

PIM: potentially inappropriate medication use.

**Table 3:** Representation of PIM use as identified according to Beers Criteria 2019.

Potentially Inappropriate Medication	No of Patients	Recommendation	Quality of Evidence	Strength of Recommendation
**1. Independent of diagnosis**				
Human insulin according to Random Blood Sugar	52	Avoid (insulin regimens containing only short- or rapid-acting insulin dosed according to current blood glucose levels without concurrent use of basal or long-acting insulin)	Moderate	Strong
Risperidone	3	Avoid, except in schizophrenia or bipolar disorder, or for short–term use as antiemetic during chemotherapy	Moderate	Strong
Clonazepam	21	Avoid	Moderate	Strong
Omeprazole	20	Avoid scheduled use for >8 weeks unless for high-risk patients (e.g., oral corticosteroids or chronic NSAID use), erosive esophagitis, Barrett’s esophagitis, pathological hypersecretory condition, or demonstrated need for maintenance treatment	High	Strong
Pantoprazole	23			
Rabeprazole	24			
Olanzapine	2	Avoid except in schizophrenia or bipolar disorder, or for short-term use as antiemetic during chemotherapy	Moderate	Strong
Zolpidem	3	Avoid	Moderate	Strong
Ketorolac	2	Avoid	Moderate	Strong
Quetiapine	2	Avoid	Moderate	Strong
Naproxen	2	Avoid	Moderate	Strong
Diclofenac	2	Avoid	Moderate	Strong
Nortriptyline	2	Avoid	High	Strong
Prazosin	6	Avoid use as an Antihypertensive	Moderate	Strong
Nitrofurantoin	3	Avoid in individuals with creatinine clearance <30 mL/min or for long term suppression	Low	Strong
Glimepiride	15	Avoid	High	Strong
Chlordiazepoxide	3	Avoid	Moderate	Strong
Clonidine	2	Avoid	Low	Strong
Trihexyphenidyl	1	Avoid	Moderate	Strong
Amitriptyline	2	Avoid	High	Strong
**2. Dependent on diagnosis**				
**Delirium**				
Clonazepam	1	Avoid	Moderate	Strong
Risperidone	1	Avoid	Moderate	Strong
**Dementia**				
Olanzapine	1	Avoid	Moderate	Strong
**3. Drug-drug interaction**				
Prazosin + Furosemide/Torsemide (alpha peripheral blocker + Loop diuretic)	4	Avoid in older women	Moderate	Strong
Hydrocortisone + ketorolac = increased risk of peptic ulcer or GI bleeding	1	Avoid; if not possible, provide gastrointestinal protection	Moderate	Strong
**4. According to creatinine clearance of the patient**				
Ranitidine < 50 mL/min	7	Reduced dose	Moderate	Strong
Enoxaparin < 30 mL/min	10	Reduced dose	Moderate	Strong
Gabapentin < 60 mL/min	4	Reduced dose	Moderate	Strong
Pregabalin < 60 mL/min	3	Reduced dose	Moderate	Strong
Spironolactone < 30 mL/min	3	Avoid	Moderate	Strong
Dabigatran < 30 mL/min	6	Avoid; dose adjustment advised when Creatine clearance (CrCl) > 30 mL/min	Moderate	Strong
Tramadol < 30 mL/min	1	Immediate release: reduce the dose Extended-release: avoid	Low	Weak
Trimethoprim-sulfamethoxazole	3	Reduced dose if CrCl 15–29 mL/min, Avoid if CrCl < 15 mL/min	Moderate	Strong

NSAID, Nonsteroidal anti-inflammatory drugs.

**Table 4:** Factors associated with the use of Potentially Inappropriate Medications.

Characteristics of Subjects	PIM (2019 Beers Criteria)		OR (95% CI)	* *p*-Value
	Present	Absent		
Gender				
Male	55 (36.6)	27 (18)	1 (reference)	
Female	56 (37.4)	12 (8)	**2.29 (1.05–4.97)**	**0.036**
Age (years)				
65–70	74 (49.5)	21 (14)	1 (reference)	
71–75	20 (13.3)	8 (5.3)	0.70 (0.27–1.83)	0.480
76–80	10 (6.7)	6 (4)	0.47 (0.15–1.45)	0.191
≥81	7 (4.6)	4 (2.6)	0.49 (0.13–1.86)	0.299
Number of comorbidities				
2	15 (10)	6 (4)	1 (reference)	
3	36 (24)	8 (5.3)	1.8 (0.53–6.08)	0.344
≥4	60 (40)	25 (16.7)	0.96 (0.33–2.75)	0.940
Length of hospital stay in days				
1–4	4 (2.6)	3 (2)	1 (reference)	
5–9	65 (43.4)	22 (14.6)	2.21 (0.45–10.68)	0.322
≥10	42 (28)	14 (9.4)	2.25 (0.44–11.30)	0.325
No of medications during a hospital stay				
1–4	3 (2)	4 (2.6)	1 (reference)	
5–9	55 (36.7)	26 (17.4)	2.82 (0.58–13.52)	0.195
≥10	53 (35.3)	9 (6)	**7.85 (1.49–41.10)**	**0.015**
Creatinine clearance (mL/min)				
<30	71 (47.4)	11 (7.3)	**12.90 (2.81–59.28)**	**0.001**
31–60	20 (13.4)	9 (6)	4.44 (0.90–21.87)	0.067
61–90	17 (11.3)	13 (8.6)	2.61 (0.54–12.48)	0.228
91–120	3 (2)	6 (4)	1 (reference)	

* *p* < 0.05 statistically significant (bolded); PIM: potentially inappropriate medication; OR: odds ratio; CI, confidence interval.
